# Effect of anemoside B4 on ameliorating cerebral ischemic/reperfusion injury

**DOI:** 10.22038/ijbms.2024.78569.16991

**Published:** 2025

**Authors:** Huizhi Fei, Xiaohuan Huang

**Affiliations:** 1 Medical college, Jiujiang University, Jiujiang, Jiangxi Province 332200, China; 2 Department of Basic Medicine, Chongqing Three Gorges Medical College, Chongqing 404100, China

**Keywords:** Anemoside B4, Apoptosis, Cerebral ischemia/reperfusion, Middle cerebral artery occlusion/reperfusion Oxygen-glucose-deprivation/re-oxygenation

## Abstract

**Objective(s):**

Anemoside B4 (AB4) is a multifunctional compound with anti-inflammatory, anti-apoptotic, antioxidant, antiviral, and autophagy-enhancing effects. However, the role of AB4 in cerebral ischemia/reperfusion injury (CIRI) remains obscure. This experiment aims to investigate the pharmacological effects of AB4 in CIRI.

**Materials and Methods:**

*In vivo*, eighty male SD rats were randomly divided into five groups: sham, MCAO/R, LD group (2.5 mg/kg), MD group (5 mg/kg), and HD group (10 mg/kg). The rats in sham and MCAO/R groups were given equal volumes of normal saline. *In vitro*, PC12 cells were divided into five groups: normal, OGD/R, OGD/R+AB4 (50 μM), OGD/R+AB4 (100 μM), and OGD/R+AB4 (200 μM). The cells were treated with hypoxia and hypoglycemia for 1.5 hr and reoxygenation for 24 hr.

**Results:**

*In vivo*, TTC and neurological scoring tests indicated that AB4 favors promoting the recovery of the brain. The histopathologic study of the brain tissues revealed that AB4 inhibited the damage of neuron cells. The TUNEL assay found that AB4 could improve cell apoptosis and prevent the brain from injury. *In vitro*, the data showed that AB4 inhibited cell damage and prevented PC12 cells from OGD/R injury, reduced IL-1β content, and increased the IL-10 level. AB4 could inhibit apoptosis of PC12 cells, down-regulate Caspase 12 and BAX expression, and up-regulate Bcl-2 expression.

**Conclusion:**

AB4 played a protective role in CIRI and could be a promising active ingredient against ischemia stroke.

## Introduction

Ischemic stork, one major reason for disability and death worldwide, is caused by inadequate cerebrovascular blood supply (1). The effective treatment is restoring the cerebrovascular blood supply. However, cerebral ischemia-reperfusion injury (CIRI) occurred accompanied by restoration of blood supply and resulted in exacerbation of tissue injury (2). The mechanism of CIRI is complex and unclear at present. Despite extensive research and development work, there is currently a lack of effective therapeutic drugs against CIRI. Thus, developing therapeutic agents to reduce reperfusion injury has become an important work and hotspot in this field. 

As a precious medical heritage, Chinese traditional medicine plays a major role in the treatment of ischemic stroke (3). In recent years, a large number of studies have shown natural compounds from Chinese medicine, such as Berberine (4), Celastrol (5), Ginsenoside (6), Tanshinone IIA (7), Resveratrol (8), have protective effects on CIRI via anti-inflammatory, anti-apoptosis, antioxidant properties. However, most of these compounds only have effects in animals and cell models. It needs more exploration in developing therapeutic candidate agents in ischemic stroke.

Anemoside B4 (AB4) is a triterpenoid saponin and monomeric natural agent extracted from *Pulsatilla chinensis.* Previous studies of AB4 have proven that AB4 exhibits anti-inflammatory, immunomodulatory, anti-apoptosis, antioxidant, antiviral, and autophagy enhancement effects (9). It has been reported that AB4 and Anemoside A3 are highly exposed *in vivo* and observed in the brain. Anemoside A3 can also improve cell injury induced by glucose deprivation in PC12 cells (10). We hypothesize that AB4 has preventive and therapeutic effects on CIRI due to its excellent anti-inflammatory and anti-apoptotic effects.

In the present study, we aim to investigate the effects of AB4 in both *in vivo* and *in* *vitro* CIRI models. Our results show that AB4 has a protective effect on CIRI.

## Materials and Methods

***Materials***

Anemoside B4 was purchased from MedChemExpress (Shanghai, China). TTC dye solution, MTT solution, TUNEL kit, and lactate dehydrogenase (LDH) kit were supplied by Wuhan Servicebio Technology. All antibodies, including Bax/Bcl2-associated X protein (BAX), Bcl-2, and Caspase12, were acquired from Wuhan Proteintech Technology. ELISA kit was purchased from Jiangsu Meibiao Biotechnology. All materials used in culturing cells were supplied by Gibco company. Sprague Dawley (SD) rats came from Chongqing Lepitt Biotechnology. 

***Animals’ model and experimental protocol***

All experiments in male SD rats were approved by the Ethics Committee of Chongqing Gorge Medical College (No: SXYZ-A-2024-01-0002). The rats, weighing 130–160 g, were reared with food and water freely and adaptively for six days. A total of 80 rats were grouped randomly: the sham group, the model group, and three AB4 groups with low, middle, and high dosages of 2.5, 5, and 10 mg/kg/d. AB4 was administrated by intraperitoneal injection for ten days. The establishment of the MCAO/R model was on the 17^th^ day. 

Rats were anesthetized and fixed in a supine position to make a 2-3 cm incision in the neck. After isolating and ligating the common carotid artery (CCA) and external carotid artery (ECA), the internal carotid artery (ICA) was separated. A loose knot was tied between the CCA and ECA for standby. The nylon suture was inserted from a 35° incision in CCA and pushed forward into ICA until the black mark points of the suture past the bifurcation. After 1.5 hr of occlusion, we withdrew the suture and ligated the CCA. Rats in the sham group were subjected to the same operation without occlusion only.

***Neurological deficit scoring***

Neurological dysfunction was analyzed by the Zea-Longa Neurological Deficit Score and Beam Balance Test on the 18^th^ day. The Zea-Longa neurological scores assayed as in the study of Peng *et al*. (11): 0 points, no neurological deficits; 1 point, inextensibility of right forelimb; 2 points, unable to walk in a straight line; 3 points, appearance of hemiplegia or tilting towards the opposite side; 4 points, unable to move or loss consciousness. 

The BBT scores follow a 6-point scale: 1 point, stand firmly on the wooden strip, without shaking, for 2 min; 2 points, stand firmly on the wooden strip, shake but do not slide down, continue for 2 min; 3 points, standing on a wooden strip, sliding to one side without falling, lasting for 2 min; 4 points, standing on a wooden strip for less than 2 min and falling off it; 5 points, tried to stand firmly on the wooden strip, but fell off within a few seconds; 6 point, no standing ability.

***2, 3, 5-Triphenyltertrazolium chloride (TTC) staining***

The TTC staining was performed to assess the infarct volume as previously described (11). Rats were sacrificed after 24 hr of reperfusion. The removed brains were kept at -20 ^°^C for half an hour and then sliced into continuous sections (1-2 mm). The sections were placed in the preheating TTC dye liquor and incubated at 37 ^°^C water bath for 15 min, then flipped over and incubated for 15 min. Then, the sections were fixed in 4% paraformaldehyde for 24 hr. The sections were photographed and analyzed using the Image J software package. The percentage infarct volume was calculated as follows: (area of non-ischemic hemisphere– area of non-ischemic tissue in the ischemic hemisphere)/area of non-ischemic hemisphere×100%.

***Hematoxylin-eosin (HE) staining and Nissl staining***

The rats were anesthetized with sodium pentobarbital and perfused with PBS and 4% paraformaldehyde. Then, the brains were removed quickly and fixed in 4% paraformaldehyde for 24 hr. After being dehydrated in a graded series of alcohols and embedded in paraffin, the brains were sliced into 5-um-thick sections for use. For HE staining, paraffin slices were immersed in hematoxylin solution for 3-5 min, rinsed with tap water for 5 min, and immersed in eosin solution for 30 sec, then soaked in gradient alcohol and dehydrated for 5 min, soaked in xylene for 30 sec, and finally sealed. For Nissl staining, the sections underwent incubation in toluidine blue solution, followed by a rinse in distilled water after 8 min, then dehydrated with gradient ethanol and treated with xylene for 5 min. Finally, microscopic examination revealed pathological damage to the cortex.

***Terminal deoxynucleotidyl transferase dUTP nick-end labeling (TUNEL) staining***

Paraffin sections were examined using a TUNEL kit to measure neuronal apoptosis. Paraffin slices were dehydrated with gradient ethanol and then immersed in 1% triton at room temperature for 20 min, raised with PBS, incubated with 50 μl equilibration buffer for 30 min, immersed in TUNEL mixture solution [56 μl mixture containing 1 μl recombinant TdT enzyme and 5 μl TMR-5-dTTP labeling Mix and 50 μl equilibration buffer] for 1.5 hr, washed, and incubated in 2 μg/ml DAPI for 10 min. The images of TUNEL staining were observed and obtained by fluorescence microscopy. 

***Cell culture and treatment***

PC12 cells grew in a 5% carbon dioxide incubator at 37 ^°^C with a complete medium. Before the experiment, AB4 was dissolved in PBS and diluted to different concentrations (50, 100, 200 μM). The cells grew to 60%, adding different concentrations of AB4, and OGD/R was performed after 24 hr growth.

***Oxygen-glucose deprivation/re-oxygenation (OGD/R) model establishment***

The cell model was performed as follows: replaced the normal medium with glucose-free DMEM medium, cultured in a three-gas incubator (5% CO_2_, 94% N_2_, 1%O_2_) for 1.5 hr, then the cells were cultured in a 5% carbon dioxide incubator for re-oxygenation with normal culture medium (89% DMEM medium, 1% penicillin/reptomycin, 10% fetal bovine serum) for 24 hr. The cell in the normal group was cultured in a 5% carbon dioxide incubator without treatment.

***3-(4,5-dimethyl-thiazol-2-yl)-2,5-diphenyl-tetrazolium bromide assay (MTT assay)***

To evaluate the cell activity after OGD/R, MTT assay was used. PC12 cells with a density of 5´10^5^ cells/ml were cultured in 96-well plates and treated with different dosages of drugs as described earlier. After OGD/R, 20 μl MTT solution was added to each well and incubated for 4 hr at 37 °C. After removing the solution in each well, 150 μl of dimethyl sulfoxide was added to dissolve the formazan and shaken for ten minutes in the dark. The optical density (OD) was measured at 490 nm by a multi-function microplate reader.

***Lactate dehydrogenase (LDH)***

To detect cell damage after drug treatment, an LDH kit was used to detect absorbance values at 450 nm using an enzyme label. PC12 cells were cultured in 6-well plates and treated with different dosages of AB4. After the OGD/R, the cell culture medium was collected into a 1.5 ml tube and centrifuged at 1000 rpm for 5 min to remove any potential cell debris or impurities. The supernatant was added to 96-well plates, worked with the prepared LDH detection solution, and then incubated in the dark for 30 min. The OD was measured by a microplate reader and used to calculate the content of LDH.

***Enzyme-linked immunosorbent assay (ELISA)***

An ELISA kit was used to detect cytokine release in a cell culture medium. The supernatant was obtained from the cell culture medium in each group. The contain of IL-1β and IL-10 were measured as instructors: setting blank holes (no samples and enzymic labeled reagents), standard holes and sample holes; adding each concentration standard products 50 μl to standard holes, the sample diluent 40 μl and sample products 10 μl to the sample holes in turn; incubating at 37 ^°^C for 30 min; washing by dd-H_2_O and then adding 50 μl of enzyme-labeled reagent to each well and incubating for 30 min; washing and adding color developer A and B 50 μl successively, mix gently, and developing color at 37 ^°^C for 15 min in the dark; adding termination liquid 50 μl to each well to terminate the reaction. The OD value of each hole was detected at 450 nm with blank hole zeroing by enzyme marker.

***Flow cytometry***

PC12 cells were treated with OGD/R and collected, washed twice with PBS, centrifuged, and detected according to the instructions of the Annexin V-FITC/PI kit. The flow cytometer was cleaned in advance, the template was selected, and the parameters were adjusted. Then, the samples to be tested were put in, and apoptosis was calculated using the values of early and late marcescence.

***Western blotting***

Bicinchoninic Acid was used to measure the concentration of the protein sample. Glue was prepared according to molecular weight size. After sample loading was completed, electrophoresis was performed, and the glue was stopped at the appropriate location. The glue was cut according to the molecular weight of the target protein. Skim milk powder was used to seal the PVDF membrane for 1-2 hr after electrorevolution. Then, the primary antibodies were prepared as anti-β-actin (1:5000), BAX (1:10000), Bcl-2 (1:1000), and Caspase 12 (1:1000). PVDF membrane was probed at 4 ^°^C overnight in primary antibody solution, and in secondary antibody (1:2000) for one hour. Finally, an ECL luminescent solution was used for development.

***Statistical analysis***

GraphPad Prism 7 was used to analyze statistical data. All data were expressed as the mean±SEM. Statistical significance was measured using normal distribution and homogeneity tests; then, one-way ANOVA was used for multiple comparisons. A value of *P* less than 0.05 denoted significance.

## Results

***AB4 reduced infarct volume and neurological deficit scores in rats subjected to MCAO/R***

**Figure 1:**
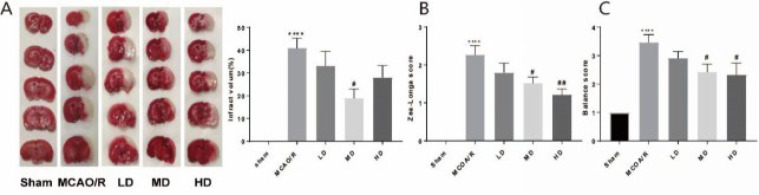
Infract volume and behavior change of rats with different dosages of AB4

To explore the function of AB4 on CIRI, infarct volume, and behavior changes were tested after MCAO/R by TTC assay, Zea-Longa scores, and Beam Balance Test. As shown in Figure 1, Compared to the sham group, infarct volume was significantly increased in the MCAO/R group; Zea-Longa scores and Beam Balance Test scores were remarkably elevated. However, an obvious reduction was observed in the middle and high dosages of the AB4 treatment group. Interestingly, these changes did not occur in the low dosage of AB4. These results suggested that AB4 could alleviate neurological impairment. 

***AB4*** ***alleviated histopathological impairment*** ***in MCAO/R rats***

**Figure 2:**
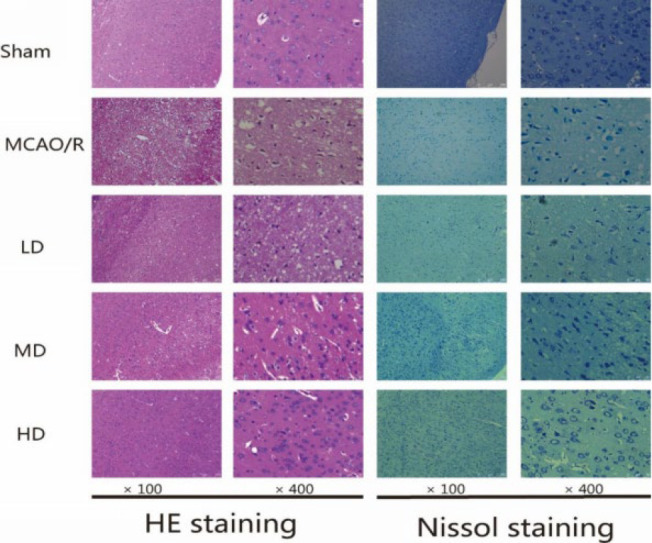
Morphology of the rat cortex with different doses of AB4

In order to investigate the histopathological changes, we observed the morphology of the rat cortex by HE staining and Nissl staining. As shown in Figure 2, in the sham group, the neuron cells exhibited a distinct and obvious outline of the nucleus without obvious edema, neurons and Nissl bodies arranged regularly in the cortex. However, in the MCAO/R group, most neurons and Nissl bodies displayed in disorder, along with a decrease in Nissl bodies, with an increase in nuclear shrinkage, pyknosis, and disappeared nucleus, with obvious edema. Compared with the MCAO/R group, the AB4 treatment group (5, 10 mg/kg) showed less swollen cells, decreased issue space, reduced edema, and more normal morphological cells arranged more regularly. These findings suggested that AB4 could alleviate brain injuries.

***AB4*** ***improved cell apoptosis in the cortex***

**Figure 3:**
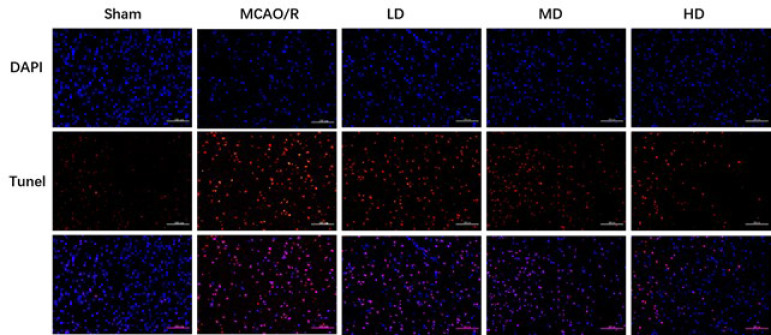
Cell apoptosis in the cortex with different dosages of AB4

Given the association between never-cell loss and apoptosis, we then explored the effect of AB4 on apoptosis. As shown in Figure 3, abundant apoptotic cells appeared in the cortex of rats subjected to MCAO/R. Following the administration of 5 mg/kg and 10 mg/kg AB4, the number of apoptotic cells was distinctly decreased. These findings and the similar trends of morphological changes further clarified that they could attenuate neuronal apoptosis induced by CIRI. 

***AB4 affected PC12 cells on cell*** ***viability***

**Figure 4:**
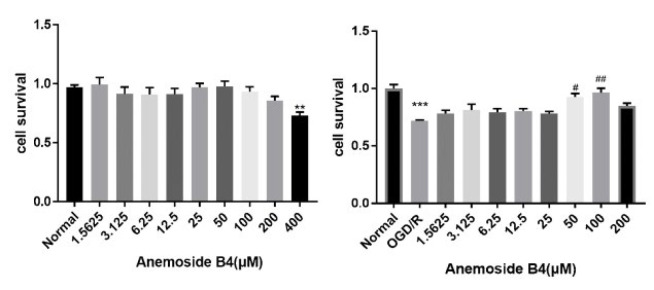
Effect of different doses of anemoside B4 (AB4) on cell viability in PC12 cells

To explore if AB4 has a pharmacological effect in PC12 cells, we first determined cell viability by MTT. We monitored that there was no significant influence on PC12 cell proliferation until the dose of AB4 was 400 μM. To further explore the effect of AB4 after OGD/R, we then chose an effective dosage by MTT under 200 μM. As shown in Figure 4, Compared to the normal group, cell survival was markedly decreased after OGD/R, and AB4 reversed the cell survival of PC12. Compared to the OGD/R group, cell survival was increased in the AB4 treatment group at the dosage of 50 μM and 100 μM. In conclusion, AB4 influenced PC12 cell survival.

***AB4 reduced PC12 cell damage***

**Figure 5:**
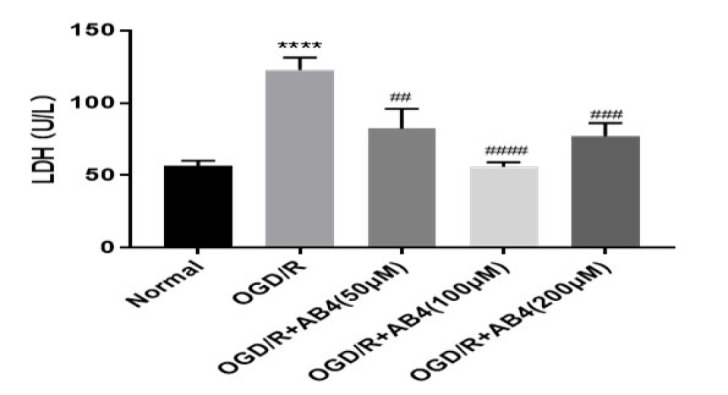
Effect of anemoside B4 (AB4) on the lactate dehydrogenase (LDH) release in PC12 cells

To explore the effect of AB4 on cell damage, we determined the activity of LDH release in cell culture fluid. As shown in Figure 5, Compared to the normal group, exposure to OGD/R notably elevated the release of LDH. Compared with the OGD/R group, the treatment of AB4 led to a significant decrease. The result suggested that AB4 alleviated cell damage.

***AB4 alleviated cell apoptosis in PC12 cells***

**Figure 6:**
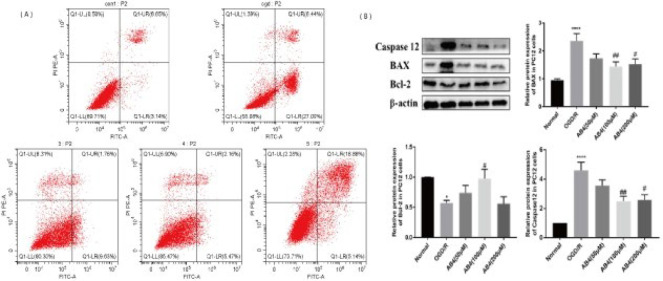
Effect of anemoside B4 (AB4) on cell apoptosis in PC12 cells

To determine whether AB4 can inhibit cell apoptosis, we measured the ratio of apoptosis, BAX, Bcl-2, and Caspase12. As shown in Figure 6 (A), exposure to OGD/R raised the ratio of apoptotic cells, and AB4 treatment could obviously reduce the percentage of apoptotic cells. Figure 6 (B) revealed that, compared with the normal group, OGD/R significantly increased the protein expression level of Caspase 12 and BAX and decreased the Bcl-2 expression level. However, the treatment of AB4 at the dosage of 100 μM and 200 μM reduced the Caspase 12 and BAX expression and elevated the level of Bcl-2 significantly. These data indicate that AB4 could improve cell apoptosis induced by OGD/R. 

***AB4 regulated inflammatory mediators in PC12 cells***

**Figure 7:**
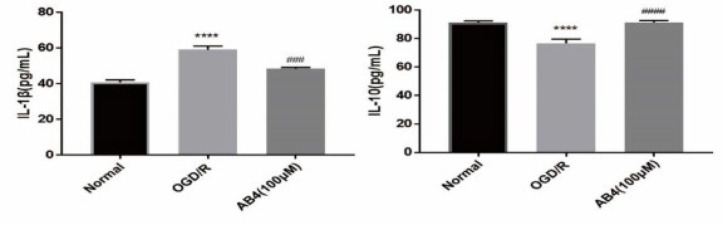
Effect of anemoside B4 (AB4) on the levels of IL-1β and IL-10 in PC12 cells

In order to explore the effect of AB4 on inflammatory factors, we assayed the change of IL-1β and IL-10. As shown in Figure 7, Compared to the normal group, the level of IL-1β was significantly increased, while the level of IL-10 was significantly decreased in the OGD/R group. Conversely, compared to the OGD/R group, the increase of IL-1β and the decrease of IL-10 were reversed effectively by 100 μM AB4 treatment. The data illustrated that AB4 affects the release of inflammatory factors.

(A) Representative TTC staining and statistical analysis of cerebral infraction rate in different groups (x̄±SEM, n=3), (B) and (C) Statistical analysis of scores tested by Zea-Longa scores and Beam Balance Test (x̄±SEM, n=9). * symbolized the statistical comparisons with the sham group, ****P<0.0001. # symbolized the statistical comparisons with MCAO/R group, ^##^*P<*0.01, #*P<*0.05

LD, low dosage (2.5 mg/kg) of AB4; MD, middle dosage (5 mg/kg) of AB4; HD, high dosage (10 mg/kg) of AB4

AB4: anemoside B4; MD: middle dosage (5mg/kg); HD: high dosage (10mg/kg)

Scale bar=250 µm and 50 µm (n=3)

LD, low dosage (2.5 mg/kg) of AB4; MD, middle dosage (5 mg/kg) of AB4; HD, high dosage (10 mg/kg) of AB4; AB4: anemoside B4

Scale bar=100 µm (n=3). LD, low dosage (2.5 mg/kg) of AB4; MD, middle dosage (5 mg/kg) of AB4; HD, high dosage (10 mg/kg) of AB4

Data were shown as x̄±SEM. * symbolized the statistical comparisons with normal group, ***P<0.001, **P<0.01. # symbolized the statistical comparisons with OGD/R group, ^##^*P<*0.01, # *P<*0.05

Data were shown as x̄±SEM. * symbolized the statistical comparisons with the normal group, ****P<0.0001. # symbolized the statistical comparisons with OGD/R group, ^####^*P<*0.0001, ^###^*P<*0.001, ^##^*P<*0.01

(A)Results of flow cytometry analysis; (B)expression of BAX, Caspase 12, and Bcl-2. Data were shown as x̄±SEM. * symbolized the statistical comparisons with normal group, ****P<0.0001, *P<0.05. # symbolized the statistical comparisons with OGD/R group, ^##^*P<*0.01, #*P<*0.05

Data were shown as x̄±SEM. * symbolized the statistical comparisons with the normal group, ****P<0.0001. # symbolized the statistical comparisons with OGD/R group, ^####^*P<*0.0001, ^###^*P<*0.001

## Discussion

In this study, MCAO/R and OGD/R were used to simulate the pathological process of CIRI* in vivo* and *in vitro* to explore the pharmacological effect of AB4. Our study showed that *in vivo *middle dosage of AB4 markedly decreased infarct volume and neurological scores, and attenuated pathomorphologic injury and apoptosis in rats significantly. We also found that *in vitro* AB4 attenuated cell injury, apoptosis, and cytokine release induced by OGD/R. These findings indicated that AB4 had potential pharmacological activity in improving brain injuries and provided experimental evidence for AB4 against CIRI. 

A variety of mechanisms were involved in CIRI, including inflammation and neuron apoptosis. 

Neuro-inflammation is involved in various stages of CIRI and could trigger neuron apoptosis, leading to neurological damage. CIRI is always followed by a marked increase in inflammatory mediators and chemiotactic proteins. Attacks of leukocytes on the ischemic area cause an inflammatory response rapidly after I/R. Pro-inflammatory cytokines increased in stroke patients and model animals (12). It is the manifestation of inflammatory responses triggered by CIRI. IL-1β is a pro-inflammatory cytokine and contributes to activating glia and infiltrating inflammatory cells, further aggravation of encephaledema and expansion of the damage to neurocytes following CIRI (13). IL-10, an anti-inflammatory cytokine, was reduced in ischemia, leading to brain damage and neuronal death (14). It has been reported that AB4 reduced the level of IL-1β and suppressed ferroptosis-mediated inflammation to alleviate arthritis pain (15). In our study, AB4 exerted its anti-inflammatory effect by inhibiting the secretion of pro-inflammatory factor IL-1β and elevating the secretion of anti-inflammatory factor IL-10 in PC12 cells. These are consistent with the results of previous studies (16-18). 

Apoptosis plays an essential role in the pathology of CIRI. In the nervous system, the mechanisms under apoptosis are complex, with multiple signaling pathways and genes involved in this process (19, 20). The Bcl-2 family and the Caspase family are key links in regulating neuronal apoptosis induced by CIRI (21, 22). Activated Caspase 12 activates downstream Caspase family members such as Caspase 3 and induces DNA fragmentation, which then results in a series of complex reactions that ultimately lead to apoptosis in CIRI (23). The most common pro-apoptotic protein, BAX, and anti-apoptotic protein, Bcl-2, in the Bcl-2 family play important roles in CIRI-induced neuronal apoptosis (24). It is well known that inhibition of neuronal apoptosis significantly improved CIRI (25). Thus, protecting neurons against delayed apoptosis is an indispensable strategy to improve post-stroke recovery. Many active compounds from natural products alleviated CIRI by inhibiting apoptosis through suppressed Bcl-2/Bax signaling pathway and activated caspase (26, 27). AB4 inhibited apoptosis while decreasing BAX, cleaved caspase-3, and cleaved PARP but increasing Bcl-2 in LPS-induced primary hepatocytes (28). Our result also showed that as a natural compound, AB4 suppressed the expression of BAX and Caspase 12 and increased the expression of Bcl-2. The data indicated that AB4 had the effect of anti-apoptosis, which is consistent with the previous results (29).

AB4, the abundance of triterpenoid saponins and the major active component of *Pulsatilla chinensis* has the potential to be an effective natural anti-inflammatory, immunoregulatory, and anti-inflammatory agent (30). AB4 has attracted significant attention. However, there are few studies regarding the role of AB4 in cerebral ischemic stroke. AB4 eventually inhibited the occurrence and development of inflammatory response by decreasing pro-inflammatory cytokines and increasing anti-inflammatory cytokines in rat endothelial cells to attenuate the leukocyte adhesion, identification, and combination of leukocytes to endothelial cells (31, 32). AB4 has treatment effects by suppressing the inflammatory reaction and anti-apoptosis in acute and chronic kidney injury, chronic obstructive pulmonary disease (COPD), lung injury, and colitis diseases (17, 33-35). Previous studies have reported that AB4 attenuated renal ischemic/reperfusion injury in rats because of the mitigation of inflammation and inhibition of apoptosis (36). Therefore, we wonder whether AB4 could improve brain injury and be a good candidate agent against CIRI. The results showed that AB4 suppressed IL-1β level and increased the level of IL-10, attenuating apoptosis *in vitro*. We also proved *in vivo* and found that AB4 alleviated neurologic behavior, improved pathological impairment, and attenuated neuron injury and apoptosis.

Our study indicated that AB4 has a protective effect on CIRI, and the anti-inflammation and anti-apoptosis effects were involved in the pharmacological mechanism. However, this study is only a preliminary exploration of the role of AB4, and its specific neuroprotective mechanism needs to be further studied in the future**. **

## Conclusion

This study demonstrated that AB4 eliminated the neurological deficit and infarct volume in the MCAO/R rats and reduced edam and pathological damage and apoptosis in MCAO/R rats. *In vitro*, AB4 attenuated the OGD/R-induced cell injury and apoptosis in PC12 cells. Our results provided experimental evidence for AB4 prevention and the treatment of CIRI and suggest that AB4 may be a candidate agent for CIRI.

## Data Availability

The data in this study can be obtained from the corresponding authors upon reasonable request.

## Declaration

We have not used any AI tools or technologies to prepare this manuscript.

## Foundation

The study was supported by the Scientific and Technological Research Program of Chongqing Municipal Education Commission (KJQN202002702) and the Traditional Chinese Medicine Science and Technology Plan Project of Jiangxi Province (2023A0239).
